# The Interaction between *Tribolium castaneum* and Mycotoxigenic *Aspergillus flavus* in Maize Flour

**DOI:** 10.3390/insects12080730

**Published:** 2021-08-14

**Authors:** Sónia Duarte, Ana Magro, Joanna Tomás, Carolina Hilário, Paula Alvito, Ricardo Boavida Ferreira, Maria Otília Carvalho

**Affiliations:** 1 Instituto Superior de Agronomia, Universidade de Lisboa, Tapada da Ajuda, 1349-017 Lisboa, Portugal; sduarte@isa.ulisboa.pt (S.D.); joannadasilva.t@hotmail.com (J.T.); carolinachilario@outlook.pt (C.H.); rbferreira@isa.ulisboa.pt (R.B.F.); motiliac@isa.ulisboa.pt (M.O.C.); 2 LEAF—Linking Landscape, Environment, Agriculture and Food, Tapada da Ajuda, 1349-017 Lisboa, Portugal; 3 National Health Institute Dr. Ricardo Jorge (INSA), 1600-609 Lisboa, Portugal; Paula.Alvito@insa.min-saude.pt; 4 Centre for Environmental and Marine Studies (CESAM), University of Aveiro, 3810-193 Aveiro, Portugal

**Keywords:** *Aspergillus flavus*, aflatoxin B1, maize flour, *Tribolium castaneum*, food safety

## Abstract

**Simple Summary:**

It is important to hold cereals in storage conditions that exclude insect pests such as the red flour beetle and fungi, especially mycotoxin-producing ones (as a few strains of *Aspergillus flavus*). This work aims to investigate the interaction between these two organisms when thriving in maize flour. It was observed that when both organisms were together, the mycotoxins detected in maize flour were far higher than when the fungi were on their own, suggesting that the presence of insects may contribute positively to fungi development and mycotoxin production. The insects in contact with the fungi were almost all dead at the end of the trials, suggesting a negative effect of the fungi growth on the insects. Both organisms interacted when in contact. This is the first study on this issue, although further investigation would benefit from clarification on the mechanisms leading to the nature of the detected interactions.

**Abstract:**

*Tribolium castaneum* is one of the most common insect pests of stored products. Its presence makes cereals more susceptible to the spread of the fungi *Aspergillus flavus*, which may produce mycotoxins. The aim of this work was to evaluate the influence of *T. castaneum* adults on the development of a mycotoxigenic *A. flavus* strain in maize flour as well as the influence of this fungus on the insects. Maize flour was exposed to *T. castaneum*, spores of *A. flavus* or to both. The results revealed an interaction between *T. castaneum* and *A. flavus* as the flour exposed to both organisms was totally colonized by the fungus whereas almost all the insects were killed. Aflatoxin B1 (AFB1) revealed a significantly higher concentration in the flour inoculated with both organisms (18.8 µg/kg), being lower when exposed only to *A. flavus*, suggesting that the presence of insects may trigger fungal development and enhance mycotoxin production. The ability of these organisms to thrive under the same conditions and the chemical compounds they release makes the interaction between them a subject of great importance to maintain the safety of stored maize. This is the first work evaluating the interaction between *T. castaneum* and *A. flavus* mycotoxin production.

## 1. Introduction

Stored products are greatly affected by both abiotic factors such as temperature and humidity and biotic factors such as insects and fungi, which may contribute to their degradation and a loss of quality and quantity. Among the biotic factors, fungi and insects are major threats and both types of organisms may either cooperate or compete to colonize the stored products, as a few authors have postulated (e.g., [1]). The presence of insects on stored food products may lead to food contamination through its body parts, excretions and secondary metabolites among others, which may be allergenic or even carcinogenic and may also lead to changes in the storage microenvironmental conditions, contributing to the spread of fungi and other microorganisms [2,3].

Several strains of the fungus *Aspergillus flavus* Link are mycotoxigenic and produce aflatoxins (AFs) that can contaminate foodstuff and become harmful to animals and humans mainly through ingestion [4,5,6,7,8]. The main aflatoxins produced by this fungus are aflatoxins belonging to the B type (B1 and B2) although a few authors have also reported the production of aflatoxins belonging to the G type (G1 and G2 [5 and references therein]). As AFB1 is carcinogenic to humans (Group 1, IARC), special attention should be dedicated to its presence in stored products that are used for human consumption in order to protect human health. The extent of fungal growth and aflatoxin production in cereal commodities depend on several biotic and abiotic factors, not only in the field but also during storage, with temperature and water availability playing key roles in these processes [6,9].

*Tribolium castaneum* (Herbst) is considered to be one of the most important key pests of stored milled grain [10]. It is also a model organism for insect development that has its genome completely sequenced [11]. The adults of this species secrete a mixture of compounds composed mainly of 1,4-benzoquinone, methyl-1,4-benzoquinone and ethyl-1,4-benzoquinone [12,13,14]. These cuticular secretions play a defensive role towards predators and microbes and a putative regulatory effect on their own population growth [15,16,17]. This insect has shown resistance to most classes of insecticides, an observation that may be partially attributed to its capacity to produce detoxification enzymes that are encoded by insecticide resistance genes such as cytochrome P450 proteins [13,18,19,20,21,22,23,24].

The interaction between insects and mycotoxigenic fungi may trigger fungi growth and mycotoxin production. Insects have been reported to be putative vectors of mycotoxigenic fungi in stored product conditions [25,26,27]. A few authors have focused mainly on the mechanical damage caused by this insect to grains and its role as a carrier of fungal spores, both contributing actively to fungal dispersion. The insects may also produce metabolic heat that is then transformed into metabolic water, thus augmenting water availability, an essential feature for mycotoxigenic fungal development [6,28,29,30]. However, it has also been shown that mycotoxins may confer an increased fungal fitness by deterring competitors or mycetophagousness [1].

Future climate change scenarios, predicting increases in temperature in several areas, indicate that food safety issues may be raised regarding not only mycotoxigenic fungi such as *A. flavus*, which are well adapted to warm weather conditions and when exposed to higher temperatures (especially combined with other abiotic factors such as water availability and carbon dioxide levels) may stimulate mycotoxin production [31,32,33], but also stored grain insect pests such as *T. castaneum*, who may increase their feeding rate or reproduction rate, which is concerning as they are vectors of mycotoxigenic fungi [34]. In addition, *T. castaneum* has also demonstrated adaptive thermal plasticity [35].

This work aimed to evaluate the possible influence of the insect presence in maize flour on the production of aflatoxins by a mycotoxigenic *A. flavus* strain as a contribution to a better understanding of this complex interaction and to the food safety of stored products.

## 2. Materials and Methods

### 2.1. Maize Zea mays L. (Poales, Poaceae) Flour Preparation

Maize collected directly from fields was stored at −4 °C and then ground and sieved to obtain maize flour. The initial maize flour moisture content and water activity were determined using adequate equipment (moisture measurement scale PMB202 ADAM (Adam Equipment, Milton Keynes, UK) and Hygrolab C1 (Rotronic, Bassersdorf, Switzerland)). The average values of the moisture content (%) and water activity (A_w_) were estimated for different interaction assays and included three replicates for each determination (*n* = 3).

### 2.2. Tribolium castaneum Mass Rearing

The red flour beetle, *Tribolium castaneum* (Herbst) (Coleoptera, Tenebrionidae), was obtained from natural populations with less than five years of rearing at the Entomology Laboratory of the Departamento de Ciências e Engenharia de Biossistemas (DCEB) of the Instituto Superior de Agronomia (ISA), University of Lisbon. The cultures were maintained at 26 °C and 65 to 70% relative humidity (RH) in a mixture of wheat flour and baker’s yeast (*Saccharomyces cerevisiae* Hansen) in a 95:5 w/w proportion, according to [36]. Mass rearing was performed as previously described [37]. The insects were maintained in a climatic chamber (Fitoclima S600, ClimaPlus 400 (ARALAB, Sintra, Portugal)), at 30 °C ± 2 °C and 70% ± 5% RH. The adults used were eight days old.

### 2.3. Aspergillus flavus Suspension

A mycotoxigenic strain of *Aspergillus flavus* Link (Eurotiales, Trichocomaceae) obtained from the Minho University Mycotheca (MUM-UMinho) was selected and maintained at 4 °C in the collection of the Laboratory of Mycology of DCEB, ISA, University of Lisbon.

Suspensions of conidia were prepared from *A. flavus*-containing potato dextrose agar (PDA) plates grown for eight days by rubbing the sporulating surface with a bent needle. After filtering through a 60 μm mesh sieve to remove debris, sterile distilled water was added to the suspension to reach a concentration of 10^7^ conidia/mL [38,39], based upon cell counts using a hemacytometer.

### 2.4. Insect/Fungi Interaction Assays

For the interaction assays, 40 g of maize flour was placed in each one of 40 glass flasks (250 mL, Depósito da Marinha Grande, Lisbon, Portugal) and autoclaved at 121 °C for 15 min to eliminate potential fungal and insect contaminations. The experimental procedure for the interaction study included different types of assays: (1) control, with maize flour only; (2) insect, with maize flour inoculated with 80 *T. castaneum* adults; (3) fungus, with maize flour inoculated with 0.5 mL of the fungal conidia suspension (see Section 2 and Section 3) and (4) mixed (insects and fungi) assay, with both 80 insect adults and 0.5 mL of the fungal conidia suspension. The flasks were sealed with a plastic lid. Ten replicates were set for each type of assay (*n* = 10).

The interaction assays were maintained in a climatic chamber at 30 °C ± 2 °C and 70% ± 5% RH for eight weeks. After this period, the living insects were counted and the level of fungal development was evaluated by a direct observation. The maize flour samples were analyzed at the laboratories of the Food and Nutrition Department, National Health Institute Dr. Ricardo Jorge (INSA), Lisbon, Portugal, for the presence of aflatoxins according to the method described in EN15851 [40] with a few modifications [41]. Two replicates of 10 g each collected from different flasks within the same type of assay were analyzed for the aflatoxin content (*n* = 2). For the mixed assay (insects and fungi), a third sample was added due to the heterogeneous aspect of the maize flour.

### 2.5. Statistical Analyses

The aflatoxin content of the different types of assays (control, insect, fungus and mixed assay) was compared for detecting significant differences (*p* < 0.05) among them. Assumptions were tested with the Bartlett test for homoscedasticity and the Shapiro–Wilk test for the normal distribution of residues. After this, the data obtained were submitted to an analysis of variance (ANOVA). If the result was considered to be significant (*p* < 0.05), Tukey’s honest significant difference test (HSD) was performed. These analyses were completed with RStudio [42] and R-3.1.2.

## 3. Results

The maize flour was incubated under appropriate conditions of 30 °C ± 2 °C and 70% ± 5% RH for eight weeks on its own (control), inoculated with *A. flavus* conidia (fungus assay), inoculated with *T. castaneum* adults (insect assay) or inoculated with both organisms (mixed assay). The mixed assay increased the mortality of the adult insects. The insect assay reared only on maize flour achieved a mortality rate of 50.0% ± 52.7% regarding the initial number of adults; in these assays, other developmental life stages were also detected and counted when present with an average number (± standard deviation) of larvae (41.4 ± 45.7), pupae (2.8 ± 4.9) and adults (123.8 ± 140.2). The mixed assay (with insects and fungi) attained a mortality rate of 99.9% ± 0.4%; it was not possible to quantify further developmental stages although it was observed that the insect adults were dead before the next generation could achieve the adult stage.

**Figure 1:**
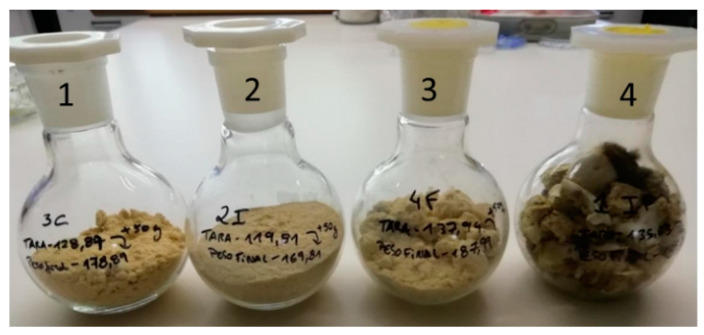
The final aspect of the interaction assays using maize flour: 1—control (containing only maize flour), 2—insect (maize flour and *Tribolium castaneum*), 3—fungus (maize flour and *Aspergillus flavus*) and 4—mixed (maize flour, *T. castaneum* and *A. flavus*).

The fungal assay showed a clearly visible fungal growth. However, a more intense fungal growth was observed in the mixed assay with the development of caking not observed in the other assays (Figure 1).

The assays conducted with insects or with fungi scored higher moisture contents and water availabilities compared with the controls although, visually, it was possible to verify that the mixed assay seemed to have an even higher moisture content and water availability (Table 1). Unfortunately, no data were available on these parameters under this study due to the development of caking during the mixed assay.

Regarding the mycotoxin analyses, the results obtained revealed the presence of aflatoxins, mainly AFB1, in the fungal (4.3 µg/kg) and mixed assay (18.8 µg/kg) followed by AFB2 although in lower concentrations in both the fungal (0.3 µg/kg) and mixed assay (0.8 µg/kg). The content of AFB1 was significantly different among the different types of assays (F = 183.1; *p* < 0.001) with the mixed assay showing a significantly higher content of aflatoxins (AFB1) than the fungal assay (*p* < 0.001) as well as the control and insect assays (*p* < 0.001 for both); the fungal assay was also considered significantly different from the control and insect assays (*p* = 0.037 for both) (Table 2). The AFB2 content was lower than AFB1 and no significant differences were found among the different types of assays (F = 2.8; *p* = 0.144).

Aflatoxin B1 (AFB1) revealed the highest concentration under the conditions of the mixed assay, exceeding the maximum legal level established for maize to be used as an ingredient in foodstuff (5 µg/kg [43]). AFB2 was also present in the mixed assay but in lower concentrations than AFB1 (0.8 µg/kg). The sum of the aflatoxin (AFB1, AFB2, AFG1, AFG2) levels (19.6 µg/kg) obtained for the mixed assay also exceeded the maximum legal values allowed for total aflatoxins in foodstuff (10 µg/kg [43]).

## 4. Discussion

The results revealed a possible interspecific competition between the red flour beetle and the mycotoxigenic fungus *A. flavus* in maize flour. The insect mortality in the interaction assays (the presence of both insect adults and fungus in the cereal flour) denoted a possible negative effect of *A. flavus* on the adults of *T. castaneum* as very high insect mortality was scored only when both organisms were present.

The fact that climate change is occurring and that aflatoxins are considered a potential health threat for cereals in Europe due to temperature and humidity increases [33,44,45,46], thus allowing optimal conditions for *A. flavus* growth, highlights the importance of assessing the potential health risks ahead for consumers and economic losses related to stored products. The increase in insect activity and fungi vectoring, together with an augmented mycotoxigenic production when both organisms are exposed to the higher temperatures recognized in future climate change scenarios, will also cause the spread of this problem to new geographical areas [33,34,47].

Aflatoxin B1, the most abundant aflatoxin detected in this study, has been shown to have a negative influence on the development and fecundity of *Ahasverus advena* Waltl, a mycetophagous insect that may also attack the food products where fungi are growing, although this insect has more tolerance to aflatoxins [48]. The significantly higher levels of aflatoxin B1 and B2 quantified in the mixed assay compared with the fungal assay may also indicate that the insects were possibly contributing to fungal favorable environmental conditions, something that has already been stated by other authors, either by contributing to the fungal dispersion or by altering the environmental conditions in terms of temperature and water availability [6,29,30,49]. For example, maize weevils seem to have an important role in the production of aflatoxins by *A. flavus* [25,27].

It is important to stress that *T. castaneum* excretes defensive secretions, benzoquinones, which compete with other organisms; namely, fungi. *Tribolium castaneum* reaches its maximum benzoquinone excretion value about 40 days after the emergence of adults [13]. Under our assay conditions, the insect adults reached 40 days of age. It therefore seems reasonable to believe that the presence of insects and their benzoquinones might have stimulated the response by *A. flavus* to produce mycotoxins (and/or other secondary defensive metabolites). However, this would need further investigation efforts as mycotoxin production is affected by several abiotic and biotic factors [1,9]. The marked negative effect of fungi on the insects corroborated the competitive nature of their relationship [1] within the conditions of the present study assays. An arms race between these two groups of organisms (insects and fungi) was hypothesized as the fungi and insects are competitors and probably their secondary metabolites are linked to the control of their competing “partners”. Based on this result, we considered that there was a competitive exclusion principle [50] when the insect was in the presence of this fungus. Further studies need to be performed to improve our understanding of the potential interaction between insects and fungi and to evaluate the presence of aflatoxins and other mycotoxins in stored products containing insects and fungi as well as their impact on the food safety of stored foodstuff.

It is important to mention that *T. castaneum* is resistant to the entomopathogenic fungi *Beauveria bassiana* (Balsamo-Crivelli) Vuillemin, reducing fungal germination and growth due to benzoquinone-defensive secretions [16,51,52,53]. Several fungi may also express benzoquinone reductases when in contact with the benzoquinone secretions of tenebrionid beetles [16]. Mycotoxins may also have harmful effects on insects [48] although all these effects should be carefully evaluated in what concerns the concentration of the metabolites (mycotoxins and benzoquinones). In addition, the conditions in terms of abiotic factors such as temperature and water availability as well as food availability should play key roles in defining the type of interactions established in each case between these organisms. Nevertheless, *T. castaneum* has an efficient detoxification system and produces benzoquinones, which are indicated to be one of the reasons for this insect’s resistance to entomopathogenic fungi [16,17,18,19,20,21,22,23,24]. The interaction between this insect and mycotoxigenic fungi is still an intriguing field of research, which may have important outcomes regarding innovative control methods of stored products associating insects and fungi. Elucidating the possible tolerance or resistance mechanism of *T. castaneum* to aflatoxins could be an important contribution to the field of novel mycotoxin control methods in the food industry, which may include the use of enzymes that promote the enzymatic degradation of mycotoxins, and new enzymes are needed [54]. This was the first work evaluating the interaction between *T. castaneum* and *A. flavus* mycotoxin production.

## Figures and Tables

**Table 1:** Average initial and final moisture content (%) and water activity (A_w_) values of different interaction assays (*n* = 3) at room temperature at the moment of A_w_ measurement (°C): control (containing only maize flour), insects (maize flour and *Tribolium castaneum*), fungi (maize flour and *Aspergillus flavus*).

Assays	Moisture Content (%)		Water Activity (A_w_) and Temperature (°C)			
	Initial	Final	Initial A_w_	Temp.	Final A_w_	Temp.
Control	8.27 ± 1.00	9.13 ± 0.30	0.60 ± 0.01	23.03 ± 0.15	0.48 ± 0.00	22.9 ± 0.06
Insects		15.30 ± 0.42			0.69 ± 0.08	22.7 ± 0.56
Fungi		21.80 ± 0.56			0.83 ± 0.03	22.8 ± 0.38

**Table 2:** The average contents (μg/kg) of aflatoxins (aflatoxin B1—AFB1; aflatoxin B2—AFB2; aflatoxin G1—AFG1; aflatoxin G2—AFG2) in the interaction assays: control (containing only maize flour, *n* = 2), insects (maize flour and *Tribolium castaneum*, *n* = 2), fungi (maize flour and *Aspergillus flavus*, *n* = 2) and insects and fungi (maize flour, *T. castaneum* and *A. flavus*, *n* = 3). Limits of detection (LOD) and quantification (LOQ) (μg/kg) are indicated for each aflatoxin analyzed. Different letters following the values of AFB1 and AFB2 in the same column indicate significantly different (*p* < 0.05) values.

Assays	AFB_1_	AFB_2_	AFG_1_	AFG_2_
Control	0.018 ± 0.006 a	<0.004 a	<0.007	<0.004
Insects	<0.011 a	<0.004 a	<0.007	<0.004
Fungi	4.306 ± 1.698 b	0.344 ± 0.139 a	<0.007	<0.004
Insects + Fungi	18.883 ± 14.160 c	0.838 ± 0.584 a	<0.007	<0.004
Limit of Detection, LOD (μg/kg)	0.011	0.004	0.007	0.004
Limit of Quantification, LOQ (μg/kg)	0.038	0.013	0.023	0.014
Maximum AFB_1_ Content for Maize in EU *	5.000			
Maximum Sum of Aflatoxins (B_1_, B_2_, G_1_, G_2)_ Content for Maize in EU *	10.000			

* Regulation (EC) 1881/2006 [43].

## Data Availability

Data sharing is not applicable to this article.
